# After-Ripening Induced Transcriptional Changes of Hormonal Genes in Wheat Seeds: The Cases of Brassinosteroids, Ethylene, Cytokinin and Salicylic Acid

**DOI:** 10.1371/journal.pone.0087543

**Published:** 2014-01-30

**Authors:** Vijaya R. Chitnis, Feng Gao, Zhen Yao, Mark C. Jordan, Seokhoon Park, Belay T. Ayele

**Affiliations:** 1 Department of Plant Science, University of Manitoba, Winnipeg, Manitoba, Canada; 2 Cereal Research Centre, Agriculture and Agri-Food Canada, Winnipeg, Manitoba, Canada; University of Nottingham, United Kingdom

## Abstract

Maintenance and release of seed dormancy is regulated by plant hormones; their levels and seed sensitivity being the critical factors. This study reports transcriptional regulation of brassinosteroids (BR), ethylene (ET), cytokinin (CK) and salicylic acid (SA) related wheat genes by after-ripening, a period of dry storage that decays dormancy. Changes in the expression of hormonal genes due to seed after-ripening did not occur in the anhydrobiotic state but rather in the hydrated state. After-ripening induced dormancy decay appears to be associated with imbibition mediated increase in the synthesis and signalling of BR, via transcriptional activation of *de-etiolated2*, *dwarf4* and *brassinosteroid signaling kinase*, and repression of *brassinosteroid insensitive 2*. Our analysis is also suggestive of the significance of increased ET production, as reflected by enhanced transcription of *1-aminocyclopropane-1-carboxylic acid oxidase* in after-ripened seeds, and tight regulation of seed response to ET in regulating dormancy decay. Differential transcriptions of *lonely guy*, *zeatin O-glucosyltransferases* and *cytokinin oxidases*, and *pseudo-response regulator* between dormant and after-ripened seeds implicate CK in the regulation of seed dormancy in wheat. Our analysis also reflects the association of dormancy decay in wheat with seed SA level and NPR independent SA signaling that appear to be regulated transcriptionally by *phenylalanine ammonia lyase*, and *whirly* and *suppressor of npr1 inducible1* genes, respectively. Co-expression clustering of the hormonal genes implies the significance of synergistic and antagonistic interaction between the different plant hormones in regulating wheat seed dormancy. These results contribute to further our understanding of the molecular features controlling seed dormancy in wheat.

## Introduction

Seed plays a vital role in the life cycle of plants as it carries genetic information from one generation to another. To this end, seed germination is an important trait playing a critical role for the establishment, growth and productivity of next generation plants. Dormancy is an adaptive mechanism through which seeds delay their germination even under optimal conditions [1]. In cereal crops such as wheat, intermediate dormancy is desirable as low level of dormancy makes seeds susceptible to preharvest sprouting that downgrades grain quality for end-use applications. Whereas, high degree of dormancy has a negative effect on the rate and uniformity of germination; ultimately causing poor seedling establishment. Most of the commercially grown wheat cultivars have low degree of dormancy and are susceptible to preharvest sprouting. This emphasizes the need to develop cultivars with moderate dormancy, for which dissection of the underlying molecular mechanisms has a paramount significance.

It is well established that the balance between two classical plant hormones, namely abscisic acid (ABA) and gibberellin (GA), is a major regulator of seed dormancy and germination [2]. However, previous studies mainly with seeds of dicot species have also implicated other plant hormones such as brassinosteroid (BR), ethylene (ET), cytokinin (CK) and salicylic acid (SA) in the regulation of these seed physiological processes [3], [4]. Brassinosteroids enhance seed germination mainly by antagonizing the inhibitory effect of ABA. When compared to that of wild type, ABA exerts stronger inhibitory effect on the germination of BR biosynthetic mutant, *det2-1* and BR-insensitive mutant, *bri1-1* seeds of Arabidopsis [5]. Consistently, inhibition of seed germination by ABA is overcome by overexpression of the BR biosynthetic gene, *DWF4* [6]. In addition, BRs reverse the non-germination phenotype of severe GA biosynthetic mutants such as *ga1-3* and the GA-insensitive mutant *sleepy1* by a mechanism different from that of GA [5], [7]. However, a recent study indicated the presence of physical interaction between repressor of *ga1-3* (RGA) and brassiazole resistant 1 (BZR1) [8], which act as negative and positive regulators of GA and BR signaling, respectively; and this might form the molecular basis of interplay between GA and BR in regulating seed dormancy and germination.

Ethylene influences several plant growth and developmental processes, from germination through senescence [9]. Previous studies with sunflower and Arabidopsis have implicated ET in seed dormancy decay [10], and promoting radicle protrusion mainly by antagonizing ABA [11]. The repression of *1-aminocyclopropane-1-carboxylic acid oxidase 1* (*ACO1*) by ABA during germination of Arabidopsis seeds, and the presence of high level of *ACO1* transcripts in the seeds of ABA-insensitive mutants [12]–[14] suggest that inhibition of germination by ABA is partly mediated through transcriptional repression of ET synthesis. In agreement with this, mutation in *ethylene response 1* (*ETR1*), a gene encoding ET receptor, led to increased seed ABA level and enhanced dormancy [15]. However, studies with cereal seeds such as barley and red rice have shown that ET is neither involved in the breaking of dormancy nor required for germination, but rather enhances radicle growth following its emergence through the pericarp [16], [17].

The role of CK in regulating seeds dormancy and germination is still unclear. Increased expression of the CK biosynthetic gene, *isopentenyltransferase8* (*IPT8*), in Arabidopsis confers ABA-insensitivity to *germination insensitive to ABA mutant 1* (*gim1*) seeds, and this is attributable to increased seed CK level and repression of ABA inducible genes including *ABI5*, a key player in seed sensitivity to ABA [4]. Consistently, CK reverses the inhibitory effect of ABA on the germination of lettuce seeds [18] and promotes the germination of wheat seeds by stimulating the GA-induced activity of α-amylase [19]. Contrary to these reports, overexpression of CK inactivating genes, *cytokinin oxidase2* (*CKX2*) and *CKX4*, and loss of function of CK receptors, *Arabidopsis histidine kinase2* (*AHK2*), *AHK3* and *ctokinin response1* (*CRE1*)*/AHK4*, leads to early germination [20].

Salicylic acid is one of the plant hormones studied extensively for its role in the local and systemic response against microbial pathogens; however, evidences involving it in the regulation of various plant growth and developmental processes including seed development and germination are emerging [21]. For example, physiological concentration of SA enhances the germination of Arabidopsis seeds by modulating the cellular level of reactive oxygen species (ROS), and thereby reducing oxidative damage [22]. This role of SA can also be associated with the regulation of seed dormancy and germination as ROS act as signaling molecules in the alleviation of seed dormancy in both monocot and dicot species [23]–[25]. Salicylic acid has also been reported to activate seed germination in wheat [26], and proteomics studies provided insights into its role in enhancing seed vigour [27]. Other studies, however, showed SA to have inhibitory effect on seed germination, such as those of maize and barley presumably by enhancing oxidative stress [28], [29], leading to the hypothesis that the effect of exogenous SA on seed germination is influenced largely by its concentration [21].

Studies that shed light on the molecular mechanisms underlying the roles of plant hormones in regulating seed dormancy and germination have been mainly focused on dicot species [3], [30]. Much less is known about these phenomena for seeds of cereal crops such as wheat. Previously, we identified specific molecular switches that mediate the roles of ABA, GA, jasmonate and auxin in regulating seed dormancy release and wheat [31]. To further our understanding with this respect, the present study explores after-ripening induced transcriptional changes of probesets related to BR, ET, CK and SA in wheat seeds in both dry and hydrated states.

## Materials and Methods

### Plant materials

Wheat cultivar “AC Domain”, which produces dormant seeds at harvest, was used as an experimental material. Immediately after harvest, a portion of seeds was stored at –80°C to maintain dormancy, while another portion was stored at room temperature and ambient relative humidity for a period of 10 months to break dormancy. Germination assays were performed under darkness at 22°C with Petri-plate system between layers of Whatman #1 paper wetted with 7 mL of sterile water. ABA treatment was performed by imbibing AR seeds with 50 µM ABA solution. Seeds were considered germinated when the coleorhiza protruded through the seed coat.

### Isolation of mRNAs and microarray analysis

mRNA samples were extracted from three independent biological replicates of dormant and after-ripened seeds at 0, 12 and 24 h after imbibition using the protocol described previously [32]. Following labeling, the mRNAs isolated from the three independent biological replicates of each sample were subjected to hybridization to the Affymetrix GeneChip Wheat Genome Array (Affymetrix). Scanning of the hybridized microarrays was performed with the Affymetrix GeneChip Operating Software. As each probeset on the Affymetrix GeneChip Wheat Genome Array is defined by 11 probe pairs, the signals obtained from all probe pairs of a given probeset were converted into a single signal value and presented in CEL file format. Adjustment of the total signal intensity in each chip was performed using the 50^th^ percentile of all measurements. The number of probesets with a ‘present’ detection call was determined by using the Affymetrix Microarray suite (MAS5) statistical algorithm. Normalization of the raw intensity data was performed with Robust Multi-array Average (RMA) methodology after which the data were log (base 2) transformed. The microarray data is available in the NCBI’s Gene Expression Omnibus database (GSE32409). Validation of the microarray data was performed with 10 selected hormonal genes (three biological and two technical replicates) using real-time quantitative RT-PCR (qRT-PCR) and the same mRNA samples subjected for microarray analysis as described before [32]. The sequences of the primers used for real-time qRT-PCR analysis are shown in Table S1.

### Identification of brassinosteroid, ethylene, cytokinin and salicylic acid related probesets

Metabolic and signaling genes of BR, ET, CK and SA were identified from the model plant Arabidopsis, and rice and other monocot species as described before [31]. Briefly, The Arabidopsis Information Resource database was searched for the target genes, and the resulting nucleotide sequences were used to search for similar sequences in the Rice Annotation Project database (http://rapdb.dna.affrc.go.jp/) [33] using a cut-off E-value of < 10^−20^. The NCBI wheat unigene build #61 (http://www.ncbi.nlm.nih.gov/UniGene/UGOrg.cgi?TAXID=4565) [34] was searched against sequences of the target genes derived from rice and other monocot species. Nucleotide sequences of wheat with a coverage length of ≥ 200 bp and an E-value of ≤ 10^−50^ were further subjected to blast search the wheat 61 K microarray platform in order to identify their respective probesets on wheat GeneChip using the search tools and criteria described previously [31]. The candidate probesets annotated by HarvEST Wheat-Chip (http://harvest.ucr.edu) [35] are shown in Table S2.

### Expression profile of brassinosteroid, ethylene, cytokinin and salicylic acid related probesets

Microarray data were analyzed by the FlexArray software (http://genomequebec.mcgill.ca/Flex-Array) [36] using analysis of variance (ANOVA) as described previously [32]. Following extraction of the log_2_ transformed signal intensities of probesets corresponding to BR, ET, CK and SA metabolism and signalling genes, log_2_ and linear fold changes in expression along with the associated *P* values were calculated between dormant and after-ripened seeds before (0 HAI) and during imbibition (12 and 24 HAI) as described before [31]. Fold changes in expression of ≥1-fold on log_2_-scale (≥2-fold on linear-scale) and probability value of *P*≤0.05 were used as cut-off values to determine statistically significant differences in expression between comparisons. While the negative fold changes indicate downregulation in the expression of genes, the positive ones reflect upregulation in each comparison under considerations.

### Co-expression clustering of hormonal probesets

Gene co-expression clustering of all the hormonal probesets (ABA, GA, Jasmonate, auxin, BR, ET, CK and SA), which exhibited statistically significant differential expression (≥2-fold and *P*≤0.05) between dormant and after-ripened seeds at any time point (Table S3), was defined by using Hierarchical Clustering (HCL) [37] of MeV (version 4.8) [38] at distance threshold of ≤ 0.25.

## Results and Discussion

### Seed germination

**Figure 1:**
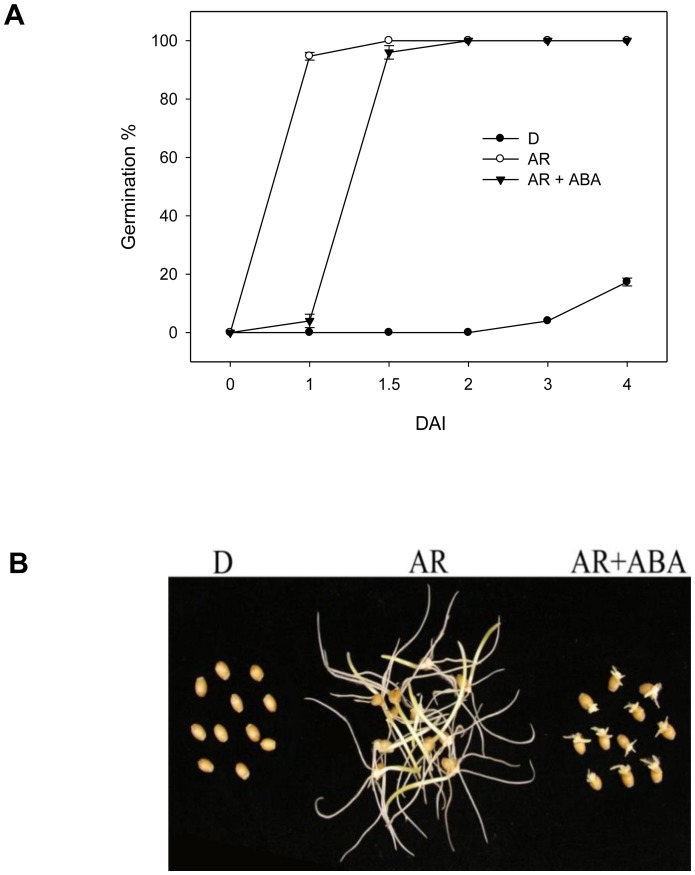
Effect of after-ripening on the release of dormancy in wheat seeds. Germination percentage of dormant (D) and after-ripened (AR) seeds imbibed in water (D and AR) and ABA (AR) over 4 days (A). Comparison of D and AR seeds following 4 day imbibition in water (D and AR) and ABA (AR). DAI, days after imbibition.

After-ripening of the dormant wheat seeds led to approximately 95% germination within 24 h of imbibition. However, the corresponding dormant seeds did not exhibit germination within the same period of imbibition, and only 11% of the dormant seeds were able to exhibit coleorhiza beyond the seed coat following 7-day imbibition. Treatment of after-ripened seeds with ABA delayed germination and inhibited seminal root growth (Figure 1).

### No transcriptional change of hormonal probesets is induced by dry after-ripening

Wheat probesets annotated as the metabolism and signaling genes of BR, ET, CK and SA were identified using bioinformatics resources based on the criteria described in the Material and Methods section (Table S2). Comparison of their expression profile between dormant and after-ripened seeds revealed that no transcriptional change is induced by dry after-ripening. Probesets annotated as ABA, GA, jasmonate and auxin metabolism and signaling genes also did not show any changes in expression due to dry after-ripening [31]. These results suggest that the transcription of hormonal genes is less likely to be an integral part of mechanisms underlying dormancy decay by dry after-ripening [39].

### After-ripening induces imbibition mediated transcriptional changes of hormonal probesets

Our transcriptomic analysis indicated that imbibition of after-ripened wheat seeds leads to changes in the transcription of specific genes related to the metabolism and signaling of BR, ET, CK and SA (Table S2). Such transcriptional changes have also been reported for those genes related to ABA, GA, jasmonate and auxin [31].

**Figure 2:**
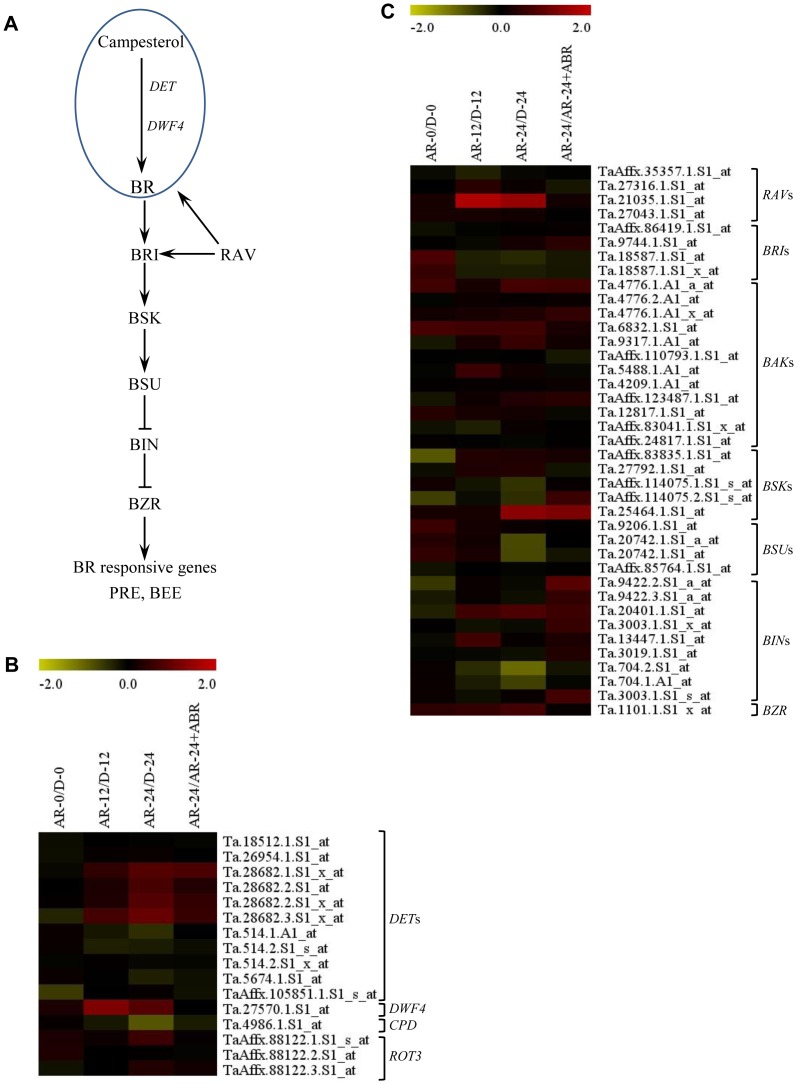
Changes in the expression of brassinosteroid (BR) metabolism and signaling genes in response to after-ripening. Simplified BR metabolic and signaling pathway in plants (A). Fold changes (log_2_-scale) in the expression of BR metabolism (B) and signaling (C) related probesets between after-ripened (AR) and dormant (D) seeds in both dry (AR-0/D-0) and imbibed states (AR-12/D-12 and AR-24/D-24), and between AR seeds imbibed without and with ABA (AR-24/AR-24+ABA). The log_2_ fold change values are shown by the negative and positive numbers on the bar, and the color scale shows upregulation (red) and downregulation (olive green) of the respective probesets. Fold changes (linear-scale) in expression and the associated *P* values are presented in Table S2. Data are means of 3 independent biological replicates. Abbreviations: *DET*, *de-etiolated*; *DWF4*, *dwarf 4*; *CPD*, *constitutive photomorphogenic dwarf*; *ROT3*, *rotundfolia 3*; *RAV*, *related to ABI3/VP1*; *BRI*, *brassinosteroid insensitive*; *BAK*, *BRI1-associated receptor kinase*; *BSK*, *BR-signaling kinases*; *BSU*, *BRI suppressor*; *BIN*, *brassinosteroid insensitive*; *BZR*, *brassinazole-resistant*.

**After-ripening induces transcriptional changes of specific brassinosteroid related probesets.** Sixteen probesets annotated as BR metabolic genes are found in the wheat genome GeneChip (Table S2), including *de-etiolated2* (*DET2*) encoding steroid 5-alpha- reductase, *constitutive photomorphogenesis and dwarfism* (*CPD*) encoding BR hydroxylase, *dwarf4* (*DWF4*) encoding steroid C-22 hydroxylase and *rotundifolia 3* (*ROT3*) encoding BR C-23 hydroxylase (Figure 2A). One of the 11 probesets of *DET2*, and a probeset annotated as *DWF4* that encodes an enzyme catalyzing a rate-limiting step in BR biosynthesis [40], exhibited upregulation (over 2-fold, *P*≤0.05) in imbibed after-ripened relative to dormant seeds (Figure 2B, Table S2). It has been shown previously that overexpression of *DWF4*, whose expression is positively correlated with bioactive BR content in plant tissues [41], [42], overcomes the inhibitory effect of ABA on germination [6], and the ABA sensitivity of *det2* seeds is higher than that of wild type [5]. Our results, thus, suggest that after-ripening activates BR synthesis during imbibition, and thereby stimulate the breakage of seed dormancy and germination through counteracting the effect of ABA.

A recent report has also indicated the contribution of *GSR*, a member of the GA stimulated transcript (GAST) family, in stimulating BR synthesis [43]. After-ripening led to upregulation (over 2-fold, *P*≤0.05) of three probesets representing *GSR* during imbibition (Table S2). Since the transcripts of probesets annotated as GA biosynthetic (*GA20ox1* and *GA3ox*) and GA responsive genes are activated in imbibing after-ripened wheat seeds [31], [32], our data might suggest the contribution of *GSR* mediated activation of BR synthesis in the regulation of wheat seed dormancy and germination.

**Figure 3:**
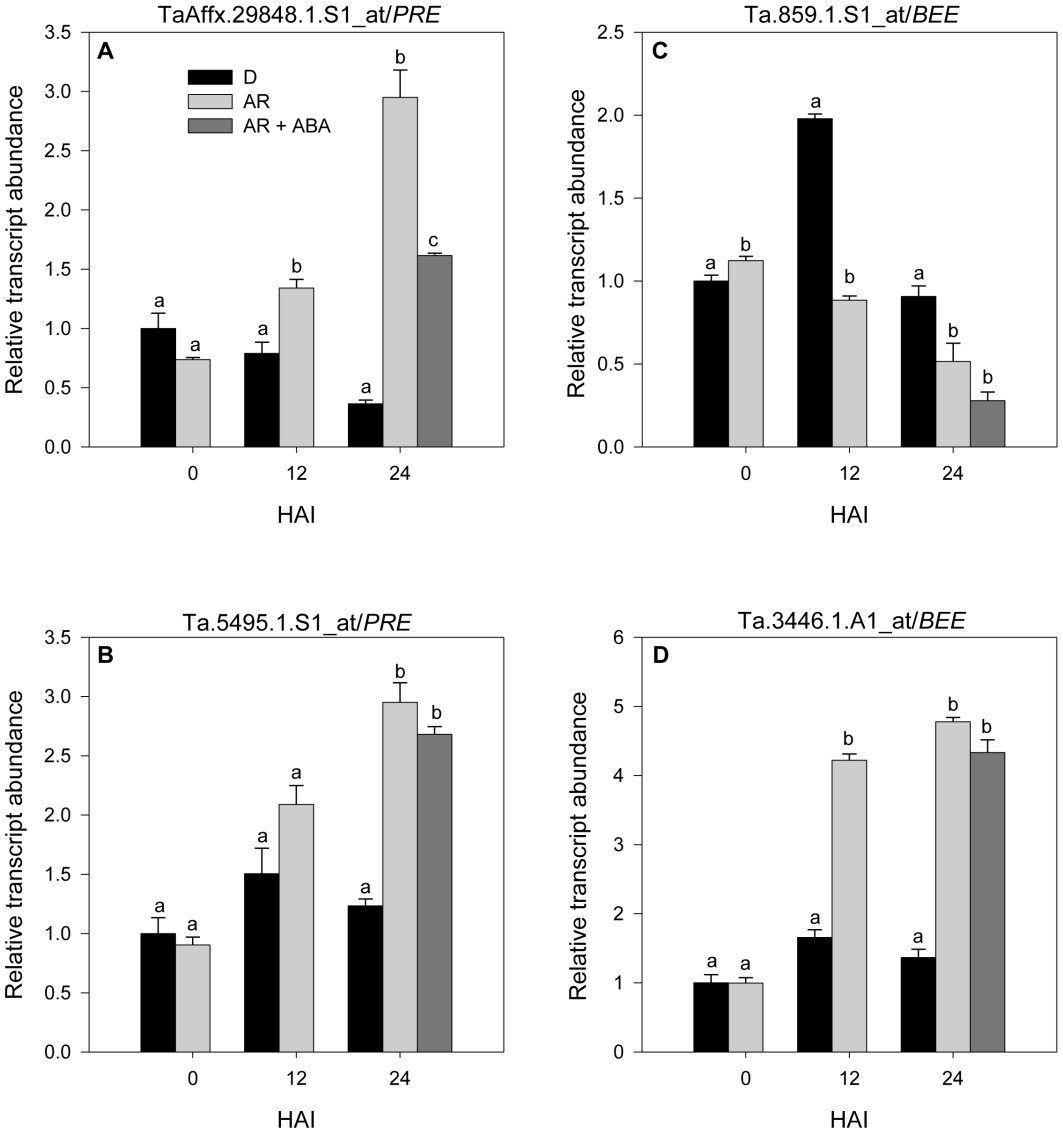
Comparison of the expression (log_2_-scale) of brassinosteroid (BR) responsive genes. Relative transcript abundance of *paclobutrazol-resistance* (*PRE*) (A, B) and *brassinosteroid enhanced expression* (*BEE*) (C, D) in dormant (D) and after-ripened (AR) seeds before imbibition (D-0 and AR-0) and during imbibition in water (D-12, AR-12, D-24, and AR-24) and ABA (AR-24+ABA). Transcript abundance was expressed relative to that in D-0 seeds, which was arbitrarily set to a value of 1. Data are means of 3 independent biological replicates ± SE. Within each imbibition time point, different letters indicate significant difference in transcript abundance between seed samples at *P*≤0.05.

Thirty nine probesets annotated as BR signalling genes are represented on Wheat GeneChip (Table S2), including *brassinosteroid insensitive 1* (*BRI1*), *regulator of the ATPase of the vacuolar membrane* (*RAV*), *BR signaling kinase* (*BSK*), *BRI1 suppressor 1* (*BSU1*), *BR-insensitive 2* (*BIN2*) and *brassiazole resistant 1* (*BZR1*) (Figure 2A). A probeset annotated as *BSK2*, which acts as a positive regulator of BR signaling, exhibited upregulation in imbibing after-ripened relative to dormant seeds, while another one annotated as *BIN2*, which acts as a negative regulator of BR signaling, was downregulated (Figure 2C, Table S2). Given that BR signaling has been implicated in the regulation of seed dormancy and germination [5], these results suggest transcriptional activation of seed response to BR leading to dormancy decay and germination. Consistently, after-ripening led to imbibition mediated transcriptional activation of probesets annotated as BR responsive genes, *paclobutrazol resistance* (*PRE*) and *BR enhanced expression* (*BEE*) (Figure 3), that are involved in cell elongation [44], [45], a process necessary for the completion of seed germination. Furthermore, our data showed transcriptional repression of *BSK2* by ABA (Figure 2C, Table S2), suggesting that one mechanism by which ABA delays wheat seed germination and inhibits seminal root growth is via repression of BR action. In support of this, ABA suppressed of the transcription of *PRE* during imbibition of after-ripened seeds.

By activating the transcription of BR biosynthetic genes, *ebisudwarf/dwarf2* (*D2*), *dwarf11* (*D11*) and *BR-deficient dwarf1/BR-C6 oxidase* (*BRD1*), and BR signaling gene, *BRI1*; the *related to ABI3/VP1* (*RAV*) *Like1* (*RAVL1*) of rice regulates not only cellular BR homeostasis but also BR sensitivity [46]–[48]. Thus, the upregulation of a probeset annotated as *RAV* in imbibing after-ripened relative to dormant seeds (over 2-fold, *P*≤0.05) (Figure 2C, Table S2) suggests the significance of coordinated regulation of BR synthesis and signaling in dormancy decay and germination of wheat seeds. Furthermore, a recent report has indicated that the antagonistic effect of BR against ABA in Arabidopsis is mediated by *mother of ft and tfl1* (*MFT*), a gene encoding a phosphatidylethanolamine, as BR cannot reverse the ABA hypersensitive and low germination phenotypes of *mft* seeds [49]. However, enhanced expression of *MFT* leads to low germination index, mimicking the inhibitory effect of ABA on germination [50]. Consistent with this, a probeset annotated as *MFT* is downregulated (over 2-fold, *P*≤0.05) in imbibing after-ripened relative to dormant seeds (Table S2), confirming that *MFT* forms an integral part of dormancy regulatory mechanisms in wheat.

**Figure 4:**
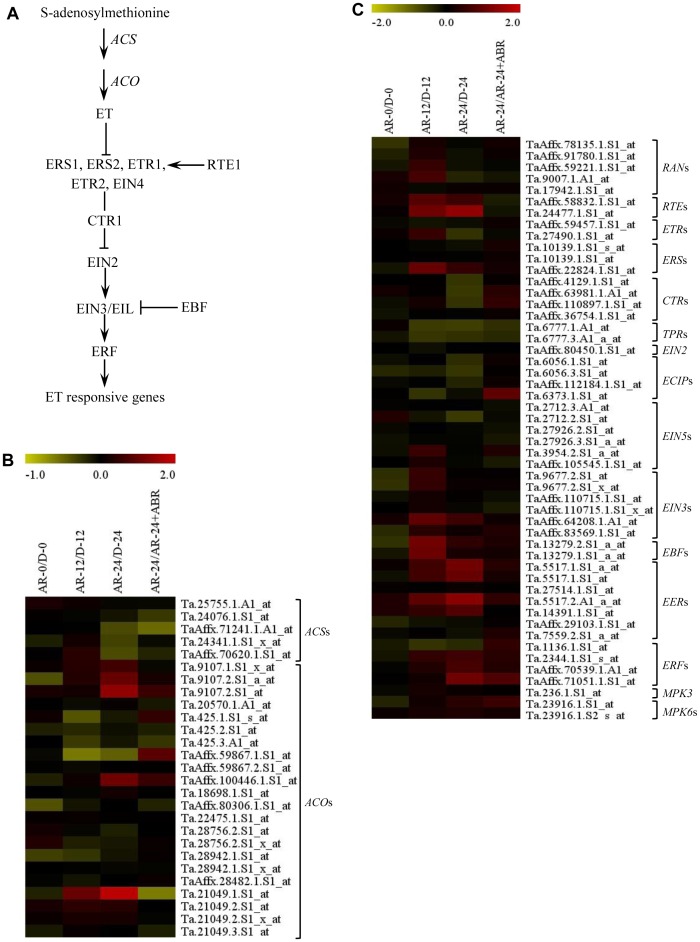
Changes in the expression of ethylene (ET) metabolism and signaling genes in response to after-ripening. Simplified ET metabolic and signaling pathway in plants (A). Fold changes (log_2_-scale) in the expression of ET metabolism (B) and signaling (C) related probesets between after-ripened (AR) and dormant (D) seeds in both dry (AR-0/D-0) and imbibed states (AR-12/D-12 and AR-24/D-24), and between AR seeds imbibed without and with ABA (AR-24/AR-24+ABA). The log_2_ fold change values are shown by the negative and positive numbers on the bar, and the color scale shows upregulation (red) and downregulation (olive green) of the respective probesets. Fold changes (linear-scale) in expression and the associated *P* values are presented in Table S2. Data are means of 3 independent biological replicates. Abbreviations: *ACS*, *1-aminocyclopropane-1-carboxylic acid synthase*; *ACO*, *1-aminocyclopropane-1-carboxylic acid oxidase*; *RAN*, *responsive-to-antagonist*; *RTE*, *reversion-to-ethylene sensitivity*; *ETR*, *ethylene response*; *ERS*, *ethylene response sensor*; *CTR*, *constitutive triple response*; *TPR*, *tetratricopeptide repeat*; *EIN2*, *ethylene insensitive 2*; *ECIP*, *EIN2 c-terminus interacting protein*; *EIN5*, *ethylene insensitive5*; *EIN3*, *ethylene insensitive 3*; *EBF*, *EIN3-binding F box protein*; *EER*, *enhanced ethylene response*; *ERF*, *ethylene-responsive element binding factor*; *MPK3*, *mitogen-activated protein kinase 3*; *MPK6*, *mitogen-activated protein kinase 6*.

**Transcriptional alteration of specific ethylene related probesets by after-ripening.** To gain insights into the role of ET in regulating dormancy and germination of wheat seeds, we investigated the differential expression of a total of 78 probesets annotated as ET metabolism and signalling genes (Table S2) between after-ripened and dormant seeds. The first committed and rate-limiting step in ethylene biosynthesis is catalyzed by 1-aminocyclopropane-1-carboxylic acid (ACC) synthase (ACS), producing ACC from S-adenosylmethionine, and the ACC is converted to ethylene by 1-aminocyclopropane-1-carboxylic acid oxidase (ACO) (Figure 4A). No probeset representing ACS was found to be differentially regulated by after-ripening (Figure 4B, Table S2). However, ethylene production is controlled by post-transcriptional mechanisms such as stabilization of the ACS protein [51], which has been shown to be regulated by BR [52]. The induction of BR biosynthetic probesets, *DET* and *DWF4*, in AR seeds might therefore imply the role of after-ripening in enhancing ACS stability through increased BR production. In contrast, four probesets corresponding to *ACO* exhibited upregulation (over 2-fold, *P*≤0.05) during imbibition of after-ripened relative to dormant seeds (Figure 4B, Table S2). Given that ET has been implicated in the breakage of seed dormancy in some species such as sunflower [10] and wild oats [53], our data might suggest after-ripening mediated induction of ET synthesis, and thereby seed dormancy release in wheat. Consistently, non-dormant seeds produce more ethylene than their dormant counterparts [3]. However, other studies have indicated that this phytohormone is not associated with dormancy decay in cereal seeds such as barley and red rice; but rather with stimulation of the germination of non-dormant seeds [16], [17]. Although ABA represses the expression of *ACO1* during germination of Arabidopsis seeds, and seeds of ABA-insensitive mutants contain high level of *ACO1* transcripts [12]–[14], no such effect of ABA was evident in wheat seeds (Figure 4B, Table S2).

Our analysis revealed that eight ET signaling probesets, ethylene response sensor1 (ERS1), reversion-to-ethylene sensitivity 1 (RTE1), EIN3-binding f box protein 1 (EBF1), enhanced ethylene response mutant 3 (EER3), ethylene-responsive element-binding protein 1 (ERF1) (Figure 4A), exhibited differential expression between imbibing after-ripened and dormant seeds (over 2-fold, P≤0.05) (Figure 4C, Table S2). In Arabidopsis, ET signalling is perceived by a family of receptors including ethylene response1 (ETR1), ETR2, ERS1, ERS2 and ethylene insensitive4 (EIN4) [54]; however, ETR1 and ERS1 play predominant roles [55]. Given that no ETR1 type receptor is present in wheat [56], the upregulation of ERS1 (over 2-fold, P≤0.05) in imbibing after-ripened seeds (Figure 4C, Table S2) suggests that transcriptional activation of ET signaling is one of the mechanisms underlying seed dormancy decay by after-ripening. Consistent with this result, probesets representing ET regulated genes such as those encoding endosperm weakening β-glucanase and chitinase B [57] are found to be upregulated during imbibition of AR seeds [32]. After-ripening also triggered imbibition induced upregulation (over 2-fold, P≤0.05) of probesets representing genes acting as negative regulators of ET signaling (Figure 4A, C; Table S2). One of these genes is RTE1, which inhibits ET signalling via positive regulation of ETR1 [58], [59]. However, RTE1 appears not to play active role in ET signaling as homolog of ETR1 does not exist in wheat [56]. Transcriptional activation of probesets representing other negative regulators of ET signaling, including EBF1 and EER3 was apparent during imbibition of after-ripened seeds, and this might suggest tight transcriptional regulation of ET signaling in wheat seeds.

**Figure 5:**
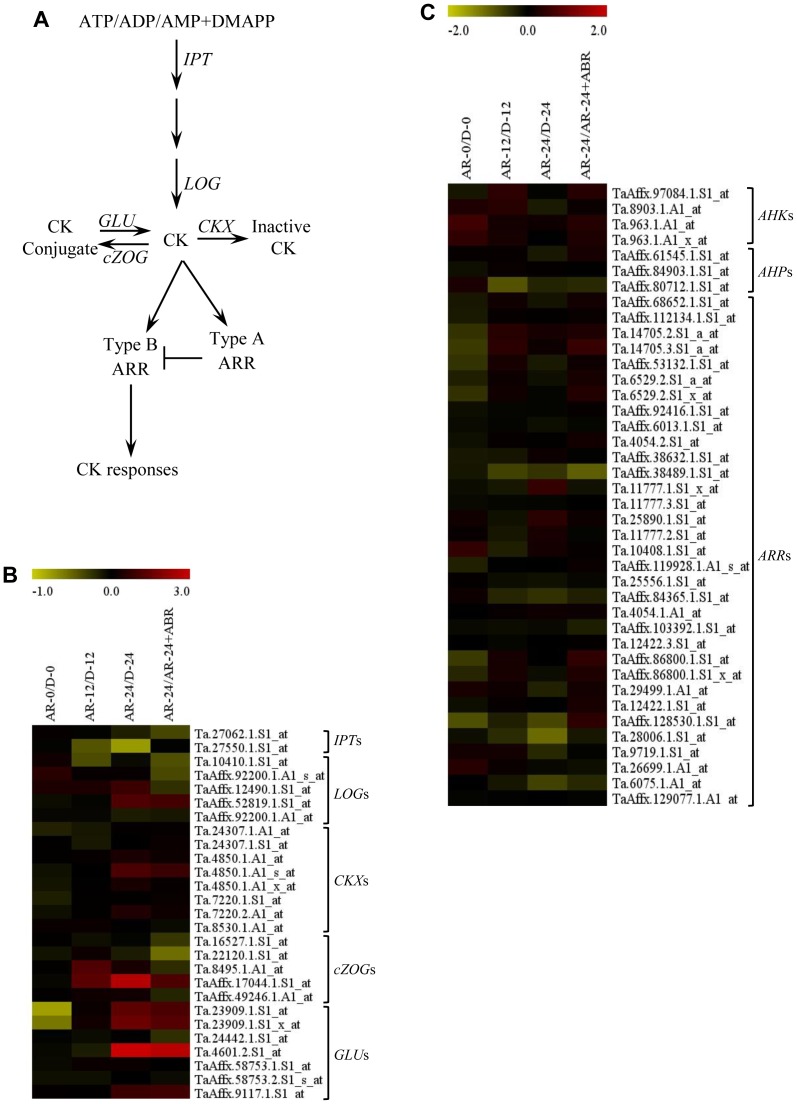
Changes in the expression of cytokinin (CK) metabolism and signaling genes in response to after-ripening. Simplified CK metabolic and signaling pathway in plants (A). Fold changes (log_2_-scale) in the expression of CK metabolism (B) and signaling (C) related probesets between after-ripened (AR) and dormant (D) seeds in both dry (AR-0/D-0) and imbibed states (AR-12/D-12 and AR-24/D-24), and between AR seeds imbibed without and with ABA (AR-24/AR-24+ABA). The log_2_ fold change values are shown by the negative and positive numbers on the bar, and the color scale shows upregulation (red) and downregulation (olive green) of the respective probesets. Fold changes (linear-scale) in expression and the associated *P* values are presented in Table S2. Data are means of 3 independent biological replicates. Abbreviations: DMAPP, dimethylallyl diphosphate; AMP, adenosine monophosphate; *IPT*, *isopentenyltransferase*; *LOG*, *lonely guy*; *CKX*, *cytokinin oxidase*; *cZOG*, *cis zeatin-o-glucoside*; *GLU*, *glucosidase*; *AHK*, *Arabidopsis histidine kinase*; *AHP*, *Arabidopsis histidine-containing phosphotransmitter*; *ARR*, *Arabidopsis response regulator*.

**After-ripening and transcriptional changes of specific cytokinin related probesets.** Based on our search criteria, the wheat genome GeneChip consists of 27 probesets annotated as genes involved in CK biosynthesis, *isopentenyl transferases* (*IPT*) and *LONELY GUY* (*LOG*); CK inactivation, *CK oxidases* (*CKX*); CK conjugation, *zeatin O-glucosyltransferases* (*ZOG*); and hydrolysis of CK conjugates, *β-glucosidases* (*GLU*) (Figure 5A, Table S2). A probeset representing *LOG* and three probesets representing *GLU* were upregulated (over 2-fold; *P*≤0.05) in imbibed after-ripened relative to dormant seeds (Figure 5B, Table S2). However, a probeset annotated as *CKX* and two probesets of *ZOG* also exhibited higher expression in after-ripened than dormant seeds during imbibition. Consistently, seeds from Arabidopsis plants overexpressing *AtCKX2* or *AtCKX4* confer early germination [20] and seed CK content declines during imbibition of non-dormant sorghum seeds [60]. Given that CK has been implicated in the breakage of seed dormancy in several species [3], our data taken together imply the importance of tight regulation of seed CK level in controlling wheat seed dormancy and germination.

Of the 40 probesets annotated as CK signaling genes (Table S2), only a probeset annotated as *APRR9*, encoding a pseudo response regulator was differentially expressed between imbibed after-ripened and dormant seeds; exhibiting over 2-fold downregulation in after-ripened relative to dormant seeds (Figure 5C; Table S2). Given that mutants of CK receptors, central players of CK signaling, exhibit early germination [20] and members of the Arabidopsis pseudo-response regulators, including APRR9, play a role in regulating physiological processes [61], our results may imply the likely involvement of *APRR* genes in regulating seed dormancy.

**Figure 6:**
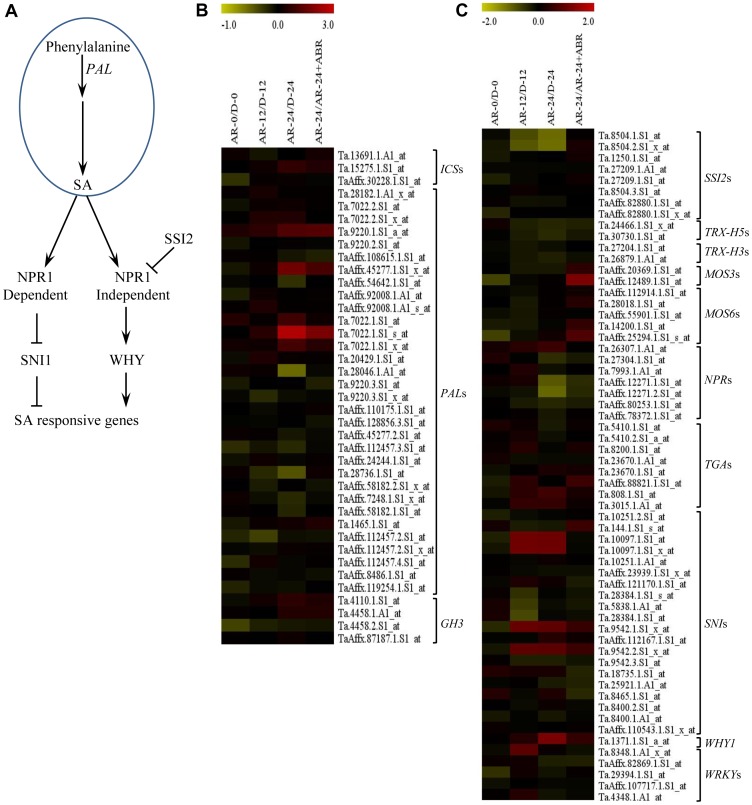
Changes in expression of salicylic acid (SA) metabolism and signaling genes in response to after-ripening. Simplified SA metabolic and signaling pathway in plants (A). Fold changes (log_2_-scale) in the expression of SA metabolism (B) and signaling (C) related probesets between after-ripened (AR) and dormant (D) seeds in both dry (AR-0/D-0) and imbibed states (AR-12/D-12 and AR-24/D-24), and between AR seeds imbibed without and with ABA (AR-24/AR-24+ABA). The log_2_ fold change values are shown by the negative and positive numbers on the bar, and the color scale shows upregulation (red) and downregulation (olive green) of the respective probesets. Fold changes (linear-scale) in expression and the associated *P* values are presented in Table S2. Data are means of 3 independent biological replicates. Abbreviations: *ICS*, *isochorismate synthase*; *PAL*, *phenylalanine ammonia-lyase*; *GH3*, *glycoside hydrolase 3*; *SSI2*, *suppressor of SA insensitive 2*; *TRX-H5*, *thioredoxin-h5*; *TRX-H3*, *thioredoxin-h3*; *MOS3*, *modifier of SNC1, 3*; *MOS6*, *modifier of SNC1, 6*; *NPR*, *Arabidopsis non-expressor of pathogenesis-related genes*; *TGA*, *TGACG motif-binding factor*; *SNI*, *suppressor of NPR1-1 inducible*; *WHY1*, *whirly 1*; *WRKY*, *WRKY DNA-binding protein*.

**Specific salicylic acid related probesets are transcriptionally regulated by after-ripening.** The biosynthesis of SA in plants contains two distinct pathways, the phenylalanine ammonia lyase (PAL)-mediated phenylalanine pathway and the isochorismate synthase (ICS)-mediated isochorismate pathway [62]. Thirty nine SA metabolic probesets representing the SA biosynthetic genes, *PAL* and *ICS*, and the SA amino acid conjugation acyl adenylase gene, *GH3* are found in the wheat genome GeneChip (Figure 6A, Table S2). Three probesets of *PAL* exhibited upregulation (over 2-fold, *P*≤0.05) in imbibed after-ripened relative to the corresponding dormant seeds, but these probesets were also repressed by ABA (Figure 6B, Table S2). These results suggest that dormancy decay and germination in wheat is associated with increased seed SA level, and the effect of ABA in delaying germination and inhibiting seminal root growth is partly mediated by transcriptional repression of SA synthesis.

Of the 60 probesets annotated as SA signalling genes (Table S2), eight probesets representing *non-expressor of PR genes* (*NPR*), *suppressor of npr1 inducible1* (*SNI*), *suppressor of SA insensitivity* (*SSI*) and *whirly* (*WHY*) (Figure 6A), exhibited differential expression (over 2-fold, *P*≤0.05) between imbibing after-ripened and dormant seeds (Figure 6C, Table S2). NPR1 has recently been identified as SA receptor, and plays a central role in SA signaling [63]. A specific probeset annotated as *NPR* exhibited downregulation (over 2 fold, *P≤*0.05) in imbibing after-ripened relative to dormant seeds (Figure 6C, Table S2), suggestive of repression of SA signaling. However, SA can also regulate the expression of its target genes independent of NPR1, and this SA signaling pathway involves *WHY*, a transcription factor acting as a positive regulator [64], [65] and *SSI2*, a gene encoding a desaturase, acting as a negative regulator (by affecting the production of a lipid derived signal required for the activation of NPR1-independent SA signalling) [66]. Our analysis revealed that a probeset representing *WHY1* and two probesets of *SSI2* are up and downregulated (over 2 fold, *P*≤0.05), respectively, in after-ripened relative to dormant seeds during imbibition (Figure 6C, Table S2). Given that SA activates seed germination in wheat [26]; our data might suggest the significance of NPR1-independent SA signalling in the regulation of dormancy decay and germination. Salicylic acid has also been shown to activate ROS scavenging enzymes in wheat [67] and enhance the germination of seeds by regulating seed ROS level [22]. Given that dormancy release by after-ripening is associated with seed ROS level [24], [25], our data might imply the contribution of NPR-independent SA signalling in the regulation of seed ROS content and thereby dormancy decay and germination in wheat.

**Figure 7:**
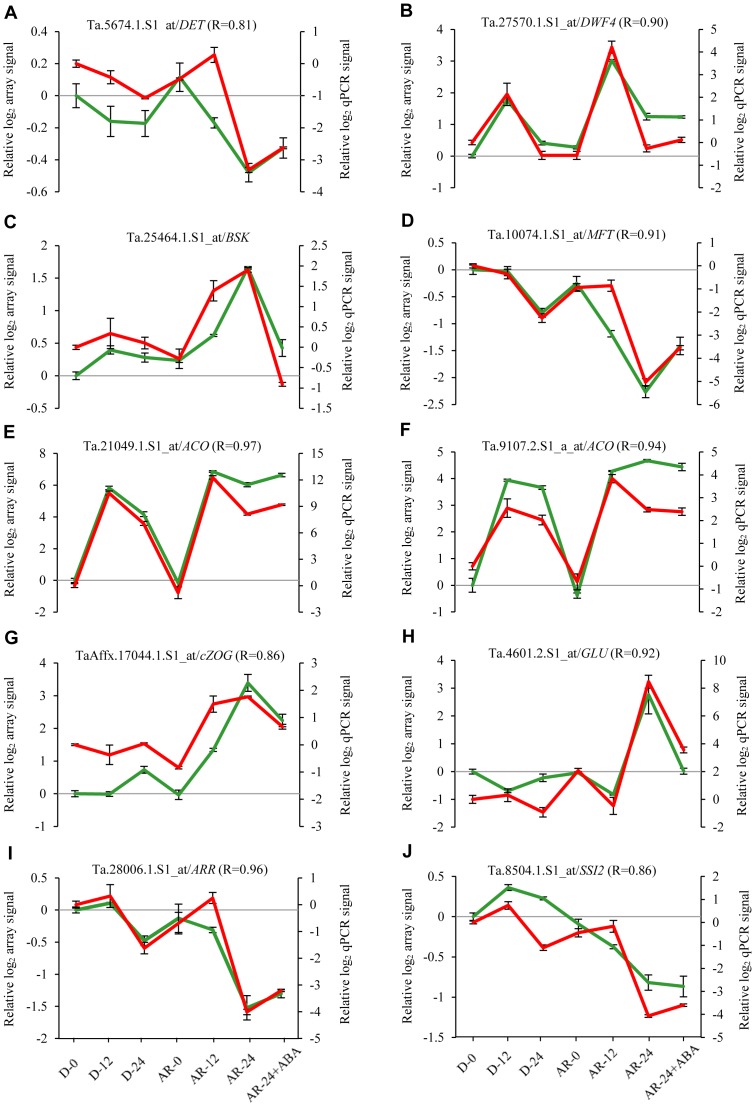
Validation of the microarray data with real-time qRT-PCR. Comparison of the microarray and qPCR data for selected probestes representing genes related to brassinosteroid (A-D), ethylene (E, F), cytokinin (G-I) and salicylic acid (J) using real time quantitative RT-PCR. Green curves in each graph represent DNA microarray data (left y-axis) while the red curves represent qPCR data (right y-axis) for both dormant (D) and after-ripened (AR) samples before imbibition (D-0 and AR-0), and after imbibition in water (D-12, D-24, AR-12 and AR-24) and ABA (AR-24+ABA). Log_2_ signal intensities for each probeset in both microarray and qPCR experiments were expressed relative to that derived from D-0 sample, which was arbirarily set to a value of 0. Data are means of 3 independent biological (and two technical) replicates ± SE. The probeset ID, the corresponding gene name and the pearson correlation coefficient (R) between the microarray and qPCR data are indicated at the top of each graph. Abbreviations: *DET*, *de-etiolated*; *DWF4*, *dwarf4; BSK*, *BR-signaling kinase*; *MFT*, *mother of FT AND TFL1*; *ACO*, *1-aminocyclopropane-1-carboxylic acid oxidase 1*; *cZOG*, *cis zeatin-o-glucoside*; *GLU*, *glucosidase*; *ARR*, *Arabidopsis response regulator*; *SSI2*, *suppressor of SA insensitive 2*.

Validation of the microarray data using real time qRT-PCR with 10 selected hormonal genes revealed a high degree of positive correlation between the array and qPCR data (Figure 7), indicating the reliability of our microarray data.

### Co-expression clustering of all hormone related probesets

**Figure 8:**
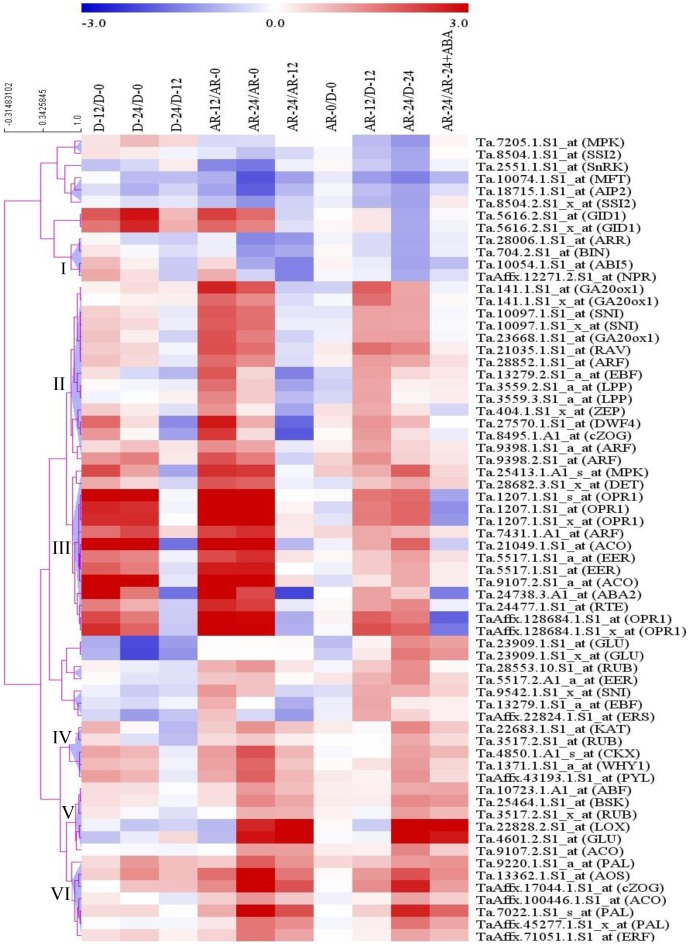
Heat map visualization of hierarchical clustering of hormone related genes. Expression values in log_2_ fold changes of genes differentially regulated (≥1-fold on log_2_-scale [≥2-fold on linear-scale], *P*≤0.05) between dormant (D) and after-ripened (AR) seeds in dry (AR-0/D-0) and hydrated (AR-12/D-12, AR24/D-24) states were used to cluster the genes on the basis of their expression pattern. The log_2_ fold change values are shown by the negative and positive numbers on the bar and the color scale shows upregulation (red) and downregulation (blue) of the respective probesets. Fold changes (linear-scale) in expression and the associated *P* values are presented in Table S3. Data are means of 3 independent biological replicates. Abbreviations: *ABI5*, *ABA insensitive 5*; *ABF*, *ABA responsive elements-binding factor; OPR1*, *12-oxophytodienoate reductase 1; AOS*, *allene oxide synthase; GA20OX1*, *GA 20 oxidase 1; GID1*, *GA insensitive dwarf 1; AIP2*, *ABI3-interacting protein 2; KAT*, *3-ketoacyl-COA thiolase; LOX*, *lipoxygenase; LPP*, *lipid phosphate phosphatase; MPK*, *mitogen-activated protein kinase; PYL*, *pyrabactin resistance-like; RUB*, *related to ubiquitin*; *SnRK*, *SNF1-related protein kinase; ZEP*, *zeaxanthin epoxidase*. Please see the legends of Figures 2, 4, 5 and 6 for the other gene abbreviations.

To gain further insights into the coordinated action of the different plant hormones in regulating after-ripening induced dormancy release and germination in wheat, we clustered all hormone related probesets that are differentially expressed between dormant and after-ripened samples (Table S3), including those reported previously [31] using hierarchical clustering method [37]. Our clustering analysis produced six groups consisting of four or more co-expressed genes related to two or more hormones (Figure 8). Given that no cluster contains probesets related to only one hormone, our result highlights the significance of synergistic and antagonistic interaction among the different hormones in regulating dormancy alleviation and germination in wheat seeds. It is likely that the genes represented by co-expressed probesets act as molecular switches underlying the interaction among the hormones represented in each cluster.

In summary, our analysis indicates imbibition induced activation and repression of specific transcriptional switches related to BR, ET, CK and SA, suggesting that seed dormancy decay by after-ripening and the subsequent germination of wheat seeds is partly mediated by changes in seed content of and response to these plant hormones. It appears from our analysis that interaction among the different plant hormones in regulating dormancy and germination of wheat seeds is transcriptionally regulated. Given that plant hormones are important regulators of seed dormancy and germination, the present study offers further insights into the molecular mechanisms underlying seed dormancy release and germination. Such knowledge is critical to develop wheat cultivars with improved tolerance to preharvest sprouting, one of the recurrent problems in the production of quality wheat.

## Supporting Information

Table S1 Primer sequences used for real-time quantitative RT-PCR analysis.
(XLSX)

Table S2 Expression of brassinosteroid, ethylene, cytokinin and salicylic acid metabolism and signaling related probesets in dormant and after-ripened seeds.
(XLSX)

Table S3 Hormone related probesets differentially expressed (≥2-fold change and *P*≤0.05) between dormant and after-ripened samples in both dry and imbibed states.
(XLSX)
